# Potential Synergy of Fluoxetine and Antibacterial Agents Against Skin and Soft Tissue Pathogens and Drug-Resistant Organisms

**DOI:** 10.3390/antibiotics13121165

**Published:** 2024-12-03

**Authors:** Samar A. Ahmed, Rondelle L. Jordan, Roslyn Rivkah Isseroff, Justin R. Lenhard

**Affiliations:** 1 Department of Clinical and Administrative Sciences, California Northstate University College of Pharmacy, Elk Grove, CA 95757, USA; samar.ahmed@cnsu.edu (S.A.A.); rondelle.jordan9893@cnsu.edu (R.L.J.); 2 Clinical Pharmacy Department, Shefa’a Al-Orman Comprehensive Cancer Center, Luxor 85863, Egypt; 3 Department of Dermatology, University of California Davis, Davis, CA 95816, USA; rrisseroff@ucdavis.edu

**Keywords:** fluoxetine, polymyxins, synergy, *Staphylococcus*, *Pseudomonas*, *Acinetobacter*, *Klebsiella*, repurposing

## Abstract

**Background/Objectives:** The feasibility of repurposing selective serotonin reuptake inhibitors as adjunctive antibacterial agents is an area of current investigation. We sought to evaluate if fluoxetine will achieve synergistic killing with relevant antibacterial drugs against skin and soft tissue pathogens and multidrug-resistant pathogens. **Methods**: The MIC of fluoxetine was determined using broth microdilution for a diverse isolate collection of 21 organisms. Checkerboard experiments were then conducted using fluoxetine and clinically relevant antibacterial drugs. If fluoxetine and an anti-infective agent achieved synergy denoted by a fractional inhibitory concentration index ≤ 0.5, then the combination was further evaluated in 24 h time-killing experiments. Synergy in time-killing experiments was defined as a ≥2 log_10_ CFU/mL reduction in fluoxetine combined with an antibacterial agent at any point in the experiment in comparison to whichever agent in the combination resulted in the lowest bacterial counts individually. **Results**: The fluoxetine MICs ranged from 64 to 128 mcg/mL for Gram-positive isolates and 8–512 mcg/mL for Gram-negative organisms. Against Gram-positive isolates, vancomycin, linezolid, clindamycin, and gentamicin failed to achieve synergy in checkerboard experiments. Levofloxacin and fluoxetine were the only combination that demonstrated synergy against a Gram-positive pathogen in both checkerboard and time-killing experiments (1/6 isolates, 16.7%). Against Gram-negative organisms, the most promising combination was fluoxetine and polymyxin B, which achieved synergistic killing in both checkerboard experiments and time-killing experiments in 12/15 isolates (80%). In comparison, fosfomycin and meropenem achieved synergy in both experiments against 6/15 (40%) and 3/15 (20%) Gram-negative isolates, respectively. **Conclusions**: The combination of fluoxetine and polymyxin B may be a potential strategy for combatting difficult-to-treat Gram-negative pathogens.

## 1. Introduction

Antimicrobial agents occupy a precarious position within the healthcare system due to their loss of effectiveness over time [1]. Following the discovery of penicillin, the spread of antimicrobial resistance was countered by the continuous generation of drugs with novel mechanisms of action or expanded scopes of coverage. With a dearth of new antimicrobial agents in the pharmaceutical pipeline, the medical community now stands on the precipice of a hypothetical post-antibiotic era characterized by a lack of therapeutic options for treating infections caused by drug-resistant pathogens [2].

Gram-negative bacteria are particularly difficult to treat due to permeability constraints imposed by the outer membrane barrier [3]. Pathogens, such as *Pseudomonas aeruginosa* and *Acinetobacter baumannii*, possess very low outer membrane permeability, and together with efflux pumps and other resistance mechanisms, the resulting phenotypes can be very difficult to treat with antimicrobials. Both *A. baumannii* and *P. aeruginosa* were identified in 2009 as “ESKAPE” pathogens by the Infectious Diseases Society of America (IDSA) due to the dwindling number of antibacterial agents that were consistently active against both pathogens [4], and the United States Centers for Diseases Control (CDC) designated carbapenem-resistant *A. baumannii* and multidrug-resistant *P. aeruginosa* as urgent and serious threats to public health, respectively [5]. *Klebsiella pneumoniae* is another Gram-negative “ESKAPE” pathogen that can develop a drug-resistant phenotype through the acquisition of resistance mechanisms against carbapenems, and such carbapenem-resistant Enterobacterale have been recognized by the CDC as an urgent threat to healthcare as well [4,5].

One strategy for treating challenging pathogens is to use a combination of two different drugs. The decision to use combination therapy is typically based on the susceptibility profile of the pathogen, the available agents, and guidance from reputable medical organizations. As an example, the IDSA recommends treating infections caused by carbapenem-resistant *A. baumannii* with sulbactam–durlobactam in combination with a carbapenem, and alternative regimens involve the combination of ampicillin–sulbactam and a second agent [6]. The IDSA also recommends ceftazidime–avibactam in combination with aztreonam as one of the preferred therapies for metallo-β-lactamase producing carbapenem-resistant Enterobacterale infections. Not only do combinations increase the likelihoodthat a pathogen is susceptible to one of the drugs used in empiric regimens, but certain agents paired together can result in more bacterial killing than the sum of each individual drug in a concept known as synergy [7].

In a related approach, several investigators have sought to expand the anti-infective armamentarium by repurposing existing drugs to potentiate the activity of antimicrobials in a combination of two or more drugs [8]. After evaluating antidepressant medications for intrinsic antimicrobial properties, selective serotonin reuptake inhibitors (SSRIs) emerged as potential candidates for further evaluation [9,10,11]. Mechanistic studies have postulated that SSRIs are capable of inhibiting bacterial efflux pumps, disrupting the integrity of the outer membrane of Gram-negative bacteria, and exerting other antibacterial effects [12,13,14,15,16]. A preliminary study with sertraline demonstrated that the drug can potentially enhance the activity of antibacterial agents against common pathogens [17], and subsequent investigations also observed that polymyxin B and sertraline achieved synergistic killing against Gram-negative organisms [13,18]. A few synergy studies were also conducted with fluoxetine, but the investigations were limited to a few strains of bacteria [19] or a single antimicrobial drug class [20].

In addition to the antimicrobial properties of fluoxetine, the SSRI is currently being evaluated for its ability to expedite wound healing. Despite the use of systemic antibacterials, many wound infections undergo a delayed healing process due to a combination of host- and pathogen-specific factors [21]. Several studies have affirmed the ability of fluoxetine to modulate host inflammatory pathways and improve healing when applied topically to wounds [22,23,24,25]. The ability of fluoxetine to expedite wound healing and adjunctively enhance the activity of antimicrobials makes the drug an ideal candidate for improving the management of skin and soft tissue infections.

The present study sought to explore the potential synergy between fluoxetine and relevant antibacterial agents against an isolate collection that included clinical isolates from skin and soft tissue infections, as well as difficult-to-treat pathogens consolidated from several sources. Following susceptibility testing using broth microdilution, synergy was initially determined using checkerboard assays. Time-killing experiments were then performed to conclude if synergistic killing was consistently observed using multiple modalities.

## 2. Results

### 2.1. Susceptibility Testing

The fluoxetine MICs for each of the studied pathogens are summarized in Table 1. With the exception of one *E. faecalis* isolate that possessed a fluoxetine MIC of 128 mcg/mL, the other five Gram-positive isolates had fluoxetine MICs of 64 mcg/mL. The fluoxetine MICs of the Gram-negative isolates varied based on the type of bacteria. Five of the *P. aeruginosa* isolates possessed a fluoxetine MIC of 256 mcg/mL, whereas two *P. aeruginosa* isolates had a fluoxetine MIC of 512 mcg/mL. Aside from one *K. pneumoniae* isolate with a fluoxetine MIC of 32 mcg/mL, the other three *K. pneumoniae* isolates had fluoxetine MICs of 128 mcg/mL. The most variability with the fluoxetine MIC was observed with the *A. baumannii* isolates, with values that ranged from 8 mcg/mL to 64 mcg/mL.

### 2.2. Checkerboard Synergy Assays

The FICI values obtained from checkerboard synergy assays are reported in Table 1. For Gram-positive organisms, the FICI values ranged from 0.38 to 1.41, with the majority of the FICIs ranging from 0.63 to 1.03. The only two drug–bug combinations that resulted in synergy were fluoxetine and cloxacillin against one of the methicillin-susceptible *S. aureus* isolates collected from a wound infection and fluoxetine and levofloxacin against the methicillin-resistant *S. aureus* strain USA 300. No synergy was observed when fluoxetine was paired with vancomycin, linezolid, or clindamycin against *S. aureus* and *E. faecalis*, and no synergy was observed for fluoxetine and ampicillin against *E. faecalis*.

Against the Gram-negative organisms, fluoxetine demonstrated the highest rate of synergy when paired with polymyxin B, achieving synergy against 13 of the 15 isolates (87%). Fosfomycin was the second most promising antibacterial agent, which resulted in synergy against 9/15 isolates (60%) when the antimicrobial was assessed with fluoxetine. Finally, meropenem was only able to achieve synergy against 4/15 isolates (27%), and neither levofloxacin nor gentamicin demonstrated a synergistic interaction with fluoxetine when assessed against the four drug-susceptible *P. aeruginosa* isolates.

### 2.3. Time-Killing Experiments

**Figure 1:**
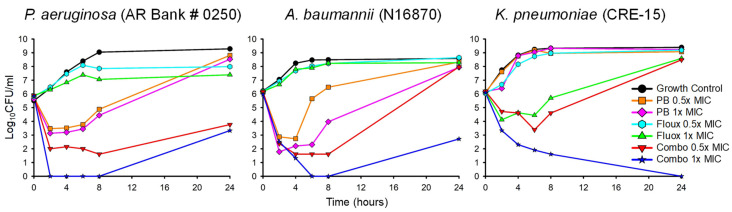
Time-killing experiments assessing the potential synergy between fluoxetine and an antimicrobial agent were conducted for multiple isolates of bacteria. Representative plots of fluoxetine and polymyxin B against different Gram-negative species are shown above. Black circle = growth of organism in the absence of drugs; orange square = exposure to a polymyxin B concentration that is half the pathogen’s MIC; pink diamond = exposure to a polymyxin B concentration equal to the pathogen’s MIC; teal hexagon = exposure to a fluoxetine concentration that is half the pathogen’s MIC; green upward triangle = exposure to a fluoxetine concentration equal to the pathogen’s MIC; red downward triangle = co-exposure to fluoxetine and polymyxin B concentrations that were equal to half their respective MICs; blue star = co-exposure to a fluoxetine and polymyxin B concentrations equal to their respective MICs.

Bug–drug duos flagged as synergistic in the checkerboard assays were further evaluated in time-killing experiments to confirm synergistic bacterial killing. Representative time-killing plots of select pathogens are displayed in Figure 1 and serve as a visual aid to conceptualize the rate and extent of bacterial killing observed during time-killing experiments. The entirety of the time-killing data is comprehensively listed in Table 2 and includes every organism that was evaluated in the time-killing experiments. All of the organisms investigated in the time-killing experiments grew more than 2 log_10_ CFU/mL between 0 and 24 h in the absence of drugs, indicating that none of the isolates possessed growth defective phenotypes. The complete set of growth control data is available in Table A1.

Of the 13 isolates identified in the checkerboard analysis of fluoxetine and polymyxin B, synergy was identified in 12 of the isolates during the time-killing experiments (92.3%), and additivity was observed for the 13th isolate. Out of the 12 isolates that experienced synergistic killing from the combination, synergy was observed at 2 h for 8/12 isolates (66.7), with an average reduction of 2.2 log_10_ CFU/mL in comparison to the agent that achieved the lowest bacterial counts individually. Synergistic killing was achieved at 4 h for all 12 of the isolates, with an average of 2.9 log_10_ CFU/mL of additional killing relative to the agent that achieved the lowest bacterial counts individually. Synergistic killing was maintained until 24 h for 10 of the 12 isolates (83.3%), with an average reduction of 4.6 log_10_ CFU/mL compared to polymyxin B or fluoxetine alone.

There was less agreement between the checkerboard assay results and time-killing experiments when fluoxetine was paired with fosfomycin. Of the nine organisms identified by the checkerboard assay, synergy was only observed in six of the isolates during time-killing experiments, with synergistic killing observed during at least four time points for four of the six isolates. Synergy was only observed at 2 h for three isolates of *P. aeruginosa*. Of the three *A. baumannii* isolates assessed in time-killing experiments, synergy was observed in one clinical isolate, additivity was observed in a separate clinical isolate, and indifference was observed against an isolate provided by the CDC and FDA Antibiotic Resistance Isolate Bank. Synergy was observed for both clinical isolates of *K. pneumoniae*, but the combination of fluoxetine and fosfomycin was antagonistic against isolate CRE-15 at four hours and then synergistic at 24 h. Antagonism was not observed for any other drug–bug combinations.

The other three antibacterial agents evaluated in time-killing experiments were meropenem, cloxacillin, and levofloxacin. The combination of meropenem and fluoxetine resulted in synergistic killing against two *K. pneumoniae* isolates and one *A. baumannii* isolate, whereas additivity was observed for the *P. aeruginosa* isolate. Although cloxacillin was synergistic against one of the methicillin-susceptible *S. aureus* isolates during checkerboard testing, the combination was only able to confer a maximum reduction of 1.4 log_10_ CFU/mL in comparison to fluoxetine or cloxacillin alone in the time-killing experiments. Lastly, the combination of levofloxacin and fluoxetine resulted in slightly less bacterial killing than fluoxetine or levofloxacin alone for timepoints at 2, 4, and 6 h, but the combination did result in 2.9 log_10_ CFU/mL of additional killing at 24 h.

## 3. Discussion

The narrow stream of novel antibacterial development has left a limited number of treatment options for acute bacterial infections. Repurposing SSRIs as adjunctive agents to use along with anti-infective agents is one strategy to increase the number of antimicrobial therapies. Here, we evaluated the potential synergy of fluoxetine with relevant antimicrobials against a mixed collection of bacterial isolates. Although the Gram-positive isolates generally possessed a lower fluoxetine MIC, levofloxacin and fluoxetine were the only combination that was synergistic against a Gram-positive isolate during checkerboard testing and time-killing experiments, and the combination was only capable of achieving synergy during a single time point of the time-killing experiments. The lower fluoxetine MICs for the Gram-positive isolates may reflect that the drug achieved higher intracellular concentrations without the need to permeate past the outer membrane of Gram-negative bacteria. In contrast, polymyxin B and fluoxetine achieved synergy against 12/15 Gram-negative isolates in both the checkerboard and time-killing experiments, whereas fosfomycin and fluoxetine only achieved synergy against 6/15 isolates.

The results of the current investigation suggest limited utility in the use of fluoxetine to synergize with systemic antibacterial agents against skin and soft tissue pathogens. *S. aureus* and *P. aeruginosa* are two of the most common bacteria isolated from chronic wound infections, and the present study included two isolates of each organism that were collected from wound infections in the clinic. The observed synergy between fluoxetine and polymyxin B against *P. aeruginosa* may indicate some benefit to the addition of fluoxetine to a topical formulation of polymyxin B. The inability of vancomycin, linezolid, clindamycin, and gentamicin to synergize against the clinical *S. aureus* isolates suggests that additional *S. aureus* killing conferred by the adjunctive use of fluoxetine is unlikely to be substantial; however, all the FICI values were 1.41 or less for the clinical *S. aureus* isolates, suggesting that fluoxetine does not interfere with the activity of the anti-staphylococcal agents. Sousa et al. investigated the potential synergy of fluoxetine with imipenem, tetracycline, and erythromycin against *S. aureus* and similarly concluded that the fluoxetine did not interfere with the action of the drugs [19]. The authors did note, however, that fluoxetine antagonized the anti-staphylococcal activity of gentamicin, but the study is limited by the use of a single *S. aureus* strain. 

In alignment with other studies, the results of the current investigation affirm the potential of SSRIs to be used as adjunctive agents against drug-resistant pathogens. The combination of fluoxetine and polymyxin B was synergistic against all of the *P. aeruginosa* isolates and the *K. pneumoniae* isolates, including pathogens that harbored carbapenemase enzymes and multidrug-resistant phenotypes. In contrast, the combination of fluoxetine and polymyxin B was only synergistic against two of four *A. baumannii* isolates in the checkerboard synergy assay, whereas synergy was only observed against a single *A. baumannii* isolate in the time-killing experiments. A study by Hussein et al. investigated the potential synergy of polymyxin B and sertraline against Gram-negative isolates with varying polymyxin susceptibilities, and the authors observed that the combination synergized against the majority of *A. baumannii* and *P. aeruginosa* isolates during checkerboard testing, but synergy was only achieved in half of the *K. pneumoniae* isolates [13]. Polymyxin B and sertraline also displayed high rates of synergy against Gram-negative organisms in a separate investigation by Otto et al. [18]. The use of an SSRI with polymyxin B may, therefore, be a promising option against difficult-to-treat Gram-negative pathogens.

The mechanism of synergy between fluoxetine and polymyxin B likely involves several different cellular processes. Previous investigators noted that fluoxetine is capable of inhibiting efflux pumps [14,15,16], which is one potential mechanism for enhancing the activity of antimicrobials that are effluxed from bacterial cells. It was also noted that fluoxetine may disrupt the outer membrane of Gram-negative bacteria and damage bacterial DNA in a process that leads to apoptosis [12]. Polymyxins, on the other hand, are well known for their ability to disrupt the integrity of the outer membrane of Gram-negative bacteria, and the disrupted barrier functionality has been posited as an explanation for why polymyxins synergize with several antibacterial classes against Gram-negative bacteria [26]. A mechanistic evaluation of sertraline and polymyxin B conducted by Hussein et al. determined that the combination of sertraline and polymyxin B together disrupted metabolic pathways involved with the synthesis and maintenance of components of the outer membrane, and electron microscopy confirmed that combination therapy led to membrane blebbing and damage to the cell envelope [13]. Taken together, the results of prior studies suggest that fluoxetine and polymyxin B are working in concert to damage the outer membrane of Gram-negative bacteria.

An important limitation of the use of fluoxetine to combat drug-resistant pathogens is the delivery of the drug to the site of infection in sufficient quantities to yield desirable anti-bacterial effects. In the present study, the MICs of fluoxetine range from 8 to 512 mcg/mL depending on the pathogen, which is in general agreement with prior investigations [19,20]. According to the manufacturer, an oral 40 mg dose of fluoxetine results in a peak plasma concentration of 15–55 ng/mL, and the majority of circulating fluoxetine is bound to proteins in human plasma [27]. In addition, the SSRI drug class carries potential adverse effects that include sexual dysfunction, seizures, bleeding, immunomodulation, and other undesirable reactions. Kromann et al. evaluated the use of oral sertraline given at four times the dose used for depression in combination with tetracycline for the treatment of tetracycline-resistant *Escherichia coli* surgical infections in chickens [28]. Animals that received sertraline and tetracycline had more severe infections than tetracycline alone, which the authors posited may be due to the immunomodulatory effects of the SSRI. In contrast, the intraperitoneal administration of fluoxetine and imipenem was superior to imipenem alone in a separate evaluation using a septic model of rats [29].

Systemic dosing of fluoxetine is, therefore, a questionable approach for the treatment of bacterial infections in humans. As an alternative, delivering fluoxetine topically as an adjuvant for wound infections is one proposed route of delivery intended to maximize concentrations of the drug at the site of infection while minimizing systemic absorption [22,23]. Several groups are currently working on modified delivery systems of fluoxetine that use microparticles, nanocapsules, and bioelectronic delivery devices [30,31,32]. Other groups have also proposed the intrathecal use of SSRIs for infections localized to the central nervous system [13], but such a paradigm is theoretical and carries significant risks.

A future direction of the current work is to better characterize different fluoxetine delivery systems and subsequently assess the feasibility of using fluoxetine as an adjunctive agent for specific sites of infection in vivo. Studies evaluating the topical use of fluoxetine to assist with wound healing may potentially quantify the concentration of fluoxetine in soft tissue. Simultaneously, collecting plasma samples from the same patients may determine how much of the topical fluoxetine circulates systemically, which will enable the concentration of the topical fluoxetine to be modulated based on the pharmacokinetics/pharmacokinetics of the topical formulation and the toxicodynamics of systemic fluoxetine. High concentrations of fluoxetine in soft tissue may help combat *P. aeruginosa*, a common pathogen in wound infections [33], as well as *A. baumannii*, a pathogen often exhibiting carbapenem resistance and occasionally associated with skin and soft tissue infections [34,35,36].

One of the most relevant sites of infection for drug-resistant Gram-negative bacteria is a lower respiratory tract infection. Given the toxicodynamic limitations of using systemic fluoxetine, an inhaled formulation of the medication may offer an attractive modality for achieving high concentrations of the drug in the epithelial lining fluid while avoiding high systemic concentrations of the drug [37]. Polymyxins are currently available in inhaled formulations, and the IDSA recommends using such medications in certain cases of ventilator-associated pneumonia caused by extensively drug-resistant pathogens [38]. Other antibacterial medications that are not approved for inhalation are occasionally nebulized to combat pathogens in the respiratory tract off-label [37], and the adjunctive use of inhaled medications for the treatment of ventilator-associated pneumonia is an area of active research [39]. If a nebulized formulation of fluoxetine is developed, the inhaled medication may be evaluated with companion drugs, such as polymyxins and fosfomycin, to combat pneumonia caused by drug-resistant pathogens.

## 4. Materials and Methods

### 4.1. Isolate Collection

A diverse isolate collection was assembled that included clinical pathogens isolated from multiple sites of infections and care centers, as well as a variety of reference strains. The 21 organisms evaluated in the current study are summarized in Table 3. Two isolates of *S. aureus* and two isolates of *P. aeruginosa* were obtained from polymicrobial wound infections at San Joaquin General Hospital, French Camp, CA. The reference strains COL and USA 300 were included to represent the methicillin-resistant *S. aureus* phenotype. The *P. aeruginosa* strain Pa01 and a second *P. aeruginosa* reference provided by the CDC and Food and Drug Administration Antimicrobial Resistance Isolate Bank were included to complement the two clinical *P. aeruginosa* isolates. *Enterococcus faecalis* was included to diversify the Gram-positive organisms evaluated in the study.

Isolates of *P. aeruginosa*, *A. baumannii*, and *Klebsiella pneumoniae* with varying drug susceptibilities were also evaluated to represent difficult-to-treat Gram-negative organisms. Three multidrug-resistant isolates of *P. aeruginosa* were provided by the CDC. Two clinical *A. baummannii* isolates that were previously characterized were included, along with reference strains obtained from the CDC or the American Type Culture Collection [40]. Lastly, four isolates of *K. pneumoniae* were previously collected from the airways of patients at Northwestern Memorial Hospital, Chicago, IL [41]. The susceptibility profile of each isolate was confirmed using broth/microbroth dilution, as outlined in the next section.

### 4.2. Susceptibility Testing

The susceptibilities of bacteria to fluoxetine were determined using broth microdilution per the specifications of the Clinical Laboratory Standards Institute [42]. Briefly, 96-well trays and Mueller–Hinton broth were used for all experiments. A fresh stock of fluoxetine (AK Scientific) was created on the day of each experiment and filter-sterilized using a 0.22 micron syringe filter. The first 8 wells in each row of the tray were filled with 100 mcl of sterile broth, and then 100 mcl of fluoxetine solution was added to the first well of each row. The contents of the wells were homogenized using a multichannel pipette, and then 100 mcl of the resulting solution was transferred to the next well in the row. The process was repeated with new pipette tips for each well until the first 8 wells in each row contained 100 mcl of broth with decreasing fluoxetine concentrations in two-fold increments. A 50 mcl suspension of 1.5 × 10^6^ CFU/mL of bacteria was then added to each well and homogenized to create an inoculum of ~5 × 10^5^ CFU/mL per well. Row 10 of the tray was filled with sterile broth to serve as a negative control, and row 12 of the tray was filled with broth and ~5 × 10^5^ CFU/mL of bacteria to serve as a positive control. Following ~24 h of incubation at 37 °C, turbidity was assessed visually and recorded. All susceptibility experiments were completed in quadruplicate, and the mode was reported as the MIC. If there were two modes, both concentrations were reported as a range.

In preparation for the checkerboard synergy assays, the MICs of clinically relevant antibacterials were determined separately using a similar methodology to fluoxetine. Vancomycin, linezolid, clindamycin, levofloxacin, gentamicin, cloxacillin, meropenem, fosfomycin, and polymyxin B powder were obtained from AK Scientific (Union City, CA, USA), and fresh stocks were prepared on the day of each experiment. Experiments involving fosfomycin included 25 mg/L of glucose-6-phosphate (Sigma Aldrich, St. Louis, MO, USA) in each well. The antibacterials assessed against each isolate were determined based on whether the organism was Gram-negative or Gram-positive and the susceptibility profile of the pathogen (Table 3). The MIC of each antibacterial was retained internally to determine the concentration of each drug needed for the checkerboard synergy assays.

### 4.3. Checkerboard Synergy Assays

As a preliminary measure of synergy, checkerboard experiments were completed using an 8 × 8 section of a 96-well tray in a manner analogous to other studies [43]. Similar to susceptibility experiments, Mueller–Hinton broth was used to achieve an inoculum of ~5 × 10^5^ CFU/mL. A gradient of fluoxetine concentrations was created vertically, and a second concentration gradient of an antimicrobial agent was created horizontally. The highest concentration assessed for each drug was 4× the observed MIC alone or the highest concentration if a range was reported from the susceptibility testing. Following ~24 h of incubation at 37 °C, visual turbidity was recorded, and the FICI was calculated for each clear well at the interface of turbidity per Equation (1). The lowest FICI value calculated from a clear well was recorded as the FICI for the experiment. Synergy was defined as an FICI value ≤ 0.5.

(1) $$FICI=\frac{{MIC}_{Fluoxetine\;in\;Combination}}{{MIC}_{Fluoxetine\;Alone}}+\frac{{MIC}_{Antibacterial\;Agent\;in\;Combination}}{{MIC}_{Antibacerial\;Agent\;Alone}}$$

### 4.4. Time-Killing Experiments

Any fluoxetine combination that was identified as potentially synergistic in the checkerboard assay was further evaluated in a time-killing experiment, as described previously [44]. In short, overnight cultures of bacteria were used to create a ~1 × 10^6^ CFU/mL inoculum in Mueller–Hinton broth. Experiments were conducted using 20 mL suspensions of bacteria, broth, and drugs contained within 50 mL conical tubes. The following reaction vessels were prepared for each investigation: no drug, half the fluoxetine MIC, the fluoxetine MIC, half the MIC of the antibacterial agent, the MIC of the antibacterial agent, the combination of fluoxetine and the antibacterial agent each at half their MIC, and the combination of fluoxetine and the antibacterial agent each at their MIC. The conical tubes were incubated in a 37 °C water bath with constant shaking at 100 RPM, and samples were collected at 0, 2, 4, 6, 8, and 24 h for serial dilution and viable plating. Following ~24 h of incubation at 37 °C, colonies were enumerated to quantify the amount of bacteria. All experiments were completed in duplicate. Synergy was defined as a ≥2 log_10_ CFU/mL reduction in one of the combination regimens at any time point in comparison to the agent that achieved the lowest bacterial counts individually, whereas additivity was defined as a ≥1 log_10_ CFU/mL comparative reduction. Antagonism was defined as ≥2 log_10_ CFU/mL of additional bacteria in one of the combination arms at any time point in comparison to the agent that achieved the lowest bacterial counts individually.

## 5. Conclusions

In closing, we observed that fluoxetine and polymyxin B achieved synergistic killing against most of the Gram-negative isolates in a collection that included drug-resistant pathogens. Fluoxetine was also capable of achieving synergistic killing against Gram-negative organisms when paired with fosfomycin, albeit to a lesser extent. The combination of fluoxetine and an antibacterial, such as polymyxin B, may be a potential strategy to combat drug-resistant pathogens if there are limited treatment options available, but future in vivo and human studies are needed to fully elucidate the clinical applicability of fluoxetine as a potentiator of antimicrobial activity. It is important to note that we were not able to identify any antibacterial agents that consistently achieved synergistic killing against Gram-positive pathogens when paired with fluoxetine. Given the pharmacokinetic and toxicodynamic challenges of administering systemic fluoxetine as an adjuvant against drug-resistant pathogens, the clinical utility of such combinations will likely depend on the delivery method of the SSRI, with topical administration or other localized routes of administration being the most desirable.

## Figures and Tables

**Table 1:** The fluoxetine MIC and FICI are reported. An FICI of 0.5 or less indicates synergy (bolded).

Organism	Isolate	Fluoxetine MIC (mcg/mL)	Fractional Inhibitory Concentration Indices *						
			VAN	LIN	CLI	AMP	CLO	LEV	GEN
*S. aureus*	Clinical 1	64	1.00	0.92	1.41	--	0.56	0.56	1.13
*S. aureus*	Clinical 2	64	0.75	1.03	1.28	--	**0.38**	1.28	0.65
*S. aureus*	COL	64	0.63	1.06	1.06	--	--	1.06	1.03
*S. aureus*	USA 300	64	0.75	1.00	1.00	--	**--**	**0.38**	1.02
*E. faecalis*	AR #0573	64	0.63	1.03	1.03	1.02	--	1.03	1.02
*E. faecalis*	AR #0671	128	1.00	0.75	0.75	1.06	--	0.63	0.75
Percentage of isolates with synergy			0%	0%	0%	0%	50%	17%	0%
Organism	Isolate	Fluoxetine MIC (mcg/mL)	Fractional Inhibitory Concentration Indices *						
			MER	FOS	POL	LEV	GEN		
*P. aeruginosa*	Pa01	256	1.13	0.63	**0.31**	1.13	1.06		
*P. aeruginosa*	AR #0258	256	0.56	0.75	**0.50**	1.06	1.06		
*P. aeruginosa*	Clinical 3	256	1.06	**0.38**	**0.19**	1.00	1.01		
*P. aeruginosa*	Clinical 4	256	0.75	**0.50**	**0.50**	0.52	0.63		
*P. aeruginosa*	AR #230	512	1.03	**0.19**	**0.19**	--	--		
*P. aeruginosa*	AR #231	512	1.02	0.63	**0.25**	--	--		
*P. aeruginosa*	AR #250	256	**0.16**	**0.28**	**0.38**	--	--		
*A. baumannii*	ATCC 19606	8	1.02	1.02	0.75	--	--		
*A. baumannii*	N16870	32	**0.50**	**0.28**	**0.38**	--	--		
*A. baumannii*	03-149.01	64	0.56	**0.19**	**0.31**	--	--		
*A. baumannii*	AR #277	64	1.03	**0.16**	1.00				
*K. pneumoniae*	CRE-15	128	**0.38**	**0.25**	**0.50**	--	--		
*K. pneumoniae*	CRE-68	32	0.75	0.56	**0.50**	--	--		
*K. pneumoniae*	CRE-118	128	**0.16**	**0.38**	**0.38**	--	--		
*K. pneumoniae*	CRE-235	128	1.13	0.75	**0.50**	--	--		
Percentage of isolates with synergy			27%	60%	87%	0%	0%		

* Vancomycin (VAN), linezolid (LIN), clindamycin (CLI), cloxacillin (CLO), levofloxacin (LEV), gentamicin (GEN), ampicillin (AMP), polymyxin B (POL), meropenem (MER), and fosfomycin (FOS) were determined based on the resistance profile of a given isolate.

**Table 2:** Following checkerboard experiments, the bacterial killing of fluoxetine and an antimicrobial agent was assessed for duos with an FICI of 0.5 or less as summarized below. A ≥2 log_10_ CFU/mL reduction in the combination relative to the agent that achieved the lowest bacterial counts individually (synergy) is in bold, whereas ≥2 log_10_ CFU/mL of additional bacteria in the combination arm (antagonism) is underlined.

Time-Killing Experiment Details					Log Reduction of Combination vs. Most Active Single Agent				
Drug	Organism	Isolate	Concentration	Interpretation	2 h	4 h	6 h	8 h	24 h
Polymyxin B (synergy in 12 isolates)	*P. aeruginosa*	Pa01	1× MIC	Synergy	**4.1**	**4.2**	**4.7**	**4.8**	**4.7**
	*P. aeruginosa*	AR #0258	1× MIC	Synergy	**3.5**	**3.3**	**2.6**	**3.0**	**6.9**
	*P. aeruginosa*	Clinical 3	0.5× MIC	Synergy	**2.0**	**3.7**	**4.6**	**5.4**	**7.5**
	*P. aeruginosa*	Clinical 4	0.5× MIC	Synergy	**3.0**	**3.9**	**4.3**	**4.8**	**7.2**
	*P. aeruginosa*	AR #230	0.5× MIC	Synergy	**4.0**	**4.4**	**5.4**	**6.0**	**2.8**
	*P. aeruginosa*	AR #231	1× MIC	Synergy	**2.1**	**2.2**	**2.4**	**3.4**	**4.1**
	*P. aeruginosa*	AR #250	1× MIC	Synergy	**2.1**	**2.2**	**2.3**	**3.1**	**5.4**
	*A. baumannii*	N16870	1× MIC	Additivity	0.5	1.2	1.8	1.4	0.4
	*A. baumannii*	03-149.01	1× MIC	Synergy	1.3	**2.9**	1.8	1.3	1.1
	*K. pneumoniae*	CRE-15	1× MIC	Synergy	1.3	**3.0**	**3.0**	**4.1**	**8.4**
	*K. pneumoniae*	CRE-68	0.5× MIC	Synergy	1.6	**2.3**	**3.1**	**3.9**	0.3
	*K. pneumoniae*	CRE-118	1× MIC	Synergy	0.8	**2.4**	**4.3**	**5.4**	**7.7**
	*K. pneumoniae*	CRE-235	1× MIC	Synergy	**2.6**	**2.1**	**2.9**	**4.2**	**3.2**
Fosfomycin(synergy in 6 isolates)	*P. aeruginosa*	Clinical 3	0.5× MIC	Synergy	**2.2**	**5.9**	**5.7**	**3.2**	−0.2
	*P. aeruginosa*	Clinical 4	1× MIC	Synergy	**4.5**	**5.5**	**5.6**	**6.0**	**4.0**
	*P. aeruginosa*	AR #230	0.5× MIC	Synergy	**5.9**	**5.6**	**5.1**	**4.3**	**7.3**
	*P. aeruginosa*	AR #250	1× MIC	Indifference	0.0	0.2	0.7	0.2	−0.2
	*A. baumannii*	N16870	1× MIC	Additivity	0.5	1.2	1.8	1.4	0.4
	*A. baumannii*	03-149.01	0.5× MIC	Synergy	0.6	1.8	**2.7**	**3.2**	0.7
	*A. baumannii*	AR #277	1× MIC	Indifference	0.3	0.1	0.1	0.2	0.3
	*K. pneumoniae*	CRE-15	0.5× MIC	Synergy/Antagonism	−1.4	1.3	−2.6	0.2	**2.1**
	*K. pneumoniae*	CRE-118	1× MIC	Synergy	0.1	**3.4**	**3.0**	**4.0**	**4.6**
Meropenem (synergy in 3 isolates)	*P. aeruginosa*	AR #250	1× MIC	Additivity	0.1	0.8	0.9	1.2	0.4
	*A. baumannii*	N16870	1× MIC	Synergy	−0.7	0.9	**2.3**	**4.0**	**5.2**
	*K. pneumoniae*	CRE-15	1× MIC	Synergy	0.3	0.6	1.8	1.9	**2.1**
	*K. pneumoniae*	CRE-118	1× MIC	Synergy	**3.0**	**2.9**	**2.5**	1.9	**2.1**
Cloxacillin	*S. aureus*	Clinical 2	1× MIC	Additivity	0.4	1.4	1.2	0.5	1.4
Levofloxacin	*S. aureus*	USA 300	1× MIC	Synergy	−0.8	−1.9	−0.5	0.0	**2.9**

**Table 3:** The 21 isolates evaluated in susceptibility testing and checkerboard synergy assays.

Organism	Isolate	Susceptibility Profile	Investigated Antibacterial Agents *
*S. aureus*	Clinical Isolate 1	Methicillin-S	VAN, LIN, CLI, LEV, GEN, CLO
*S. aureus*	Clinical Isolate 2	Methicillin-S	VAN, LIN, CLI, LEV, GEN, CLO
*S. aureus*	COL	Methicillin-R	VAN, LIN, CLI, LEV, GEN
*S. aureus*	USA 300	Methicillin-R	VAN, LIN, CLI, LEV, GEN
*E. faecalis*	AR Bank #0573	Ampicillin-S	VAN, LIN, AMP, LEV, GEN
*E. faecalis*	AR Bank #0671	Ampicillin-S	VAN, LIN, AMP, LEV, GEN
*P. aeruginosa*	Pa01	Drug-S	MER, FOS, POL, LEV, GEN
*P. aeruginosa*	AR Bank #0258	Drug-S	MER, FOS, POL, LEV, GEN
*P. aeruginosa*	Clinical Isolate 3	Drug-S	MER, FOS, POL, LEV, GEN
*P. aeruginosa*	Clinical Isolate 4	Drug-S	MER, FOS, POL, LEV, GEN
*P. aeruginosa*	AR Bank #230	Multidrug-R	MER, FOS, POL
*P. aeruginosa*	AR Bank #231	Multidrug-R	MER, FOS, POL
*P. aeruginosa*	AR Bank #0250	Multidrug-R	MER, FOS, POL
*A. baumannii*	ATCC 19606	Drug-S	MER, FOS, POL
*A. baumannii*	N16870 (clinical)	Multidrug-R	MER, FOS, POL
*A. baumannii*	03-149.01 (clinical)	Multidrug-R	MER, FOS, POL
*A. baumannii*	AR Bank #277	Multidrug-R	MER, FOS, POL
*K. pneumoniae*	CRE-15 (clinical)	Multidrug-R	MER, FOS, POL
*K. pneumoniae*	CRE-68 (clinical)	Multidrug-R	MER, FOS, POL
*K. pneumoniae*	CRE-118 (clinical)	Multidrug-R	MER, FOS, POL
*K. pneumoniae*	CRE-235 (clinical)	Multidrug-R	MER, FOS, POL

* Vancomycin (VAN), linezolid (LIN), clindamycin (CLI), cloxacillin (CLO), levofloxacin (LEV), gentamicin (GEN), ampicillin (AMP), polymyxin B (POL), meropenem (MER), and fosfomycin (FOS) were determined based on the resistance profile of a given isolate.

## Data Availability

Data are contained within the article.

## Appendix A

**Table A1:** The average concentration of bacteria contained in duplicated growth control arms is listed for each time-killing experiment.

			Average Concentration of Bacteria in Growth Controls (Log_10_ CFU/mL)					
Drug	Organism	Isolate	0 h	2 h	4 h	6 h	8 h	24 h
Controls for experiments that evaluated polymyxin B	*P. aeruginosa*	Pa01	5.9	6.8	8.2	8.7	9.1	9.7
	*P. aeruginosa*	AR #0258	6.2	7.0	8.5	8.8	9.2	9.5
	*P. aeruginosa*	Clinical 3	6.0	6.9	8.3	9.0	9.1	9.6
	*P. aeruginosa*	Clinical 4	6.2	7.1	8.3	8.7	8.9	9.7
	*P. aeruginosa*	AR #230	6.0	6.9	8.0	8.8	8.8	9.3
	*P. aeruginosa*	AR #231	5.8	6.5	8.0	8.6	8.9	9.7
	*P. aeruginosa*	AR #250	5.5	6.4	7.6	8.4	9.0	9.3
	*A. baumannii*	N16870	6.2	7.0	8.2	8.5	8.5	8.6
	*A. baumannii*	03-149.01	6.2	7.4	7.6	7.9	8.2	8.7
	*K. pneumoniae*	CRE-15	6.1	7.7	8.8	9.2	9.3	9.4
	*K. pneumoniae*	CRE-68	6.0	7.5	8.7	9.1	9.2	9.3
	*K. pneumoniae*	CRE-118	6.2	8.0	9.0	9.2	9.3	9.6
	*K. pneumoniae*	CRE-235	5.8	7.7	9.0	9.5	9.4	9.5
Controls for experiments that evaluated fosfomycin	*P. aeruginosa*	Clinical 3	5.8	6.8	8.2	8.7	9.1	9.6
	*P. aeruginosa*	Clinical 4	6.5	7.2	8.5	8.5	8.9	9.6
	*P. aeruginosa*	AR #230	5.9	6.9	7.7	8.7	8.9	9.4
	*P. aeruginosa*	AR #250	5.6	6.0	7.1	7.2	7.7	9.0
	*A. baumannii*	N16870	5.7	7.6	8.3	8.4	8.5	8.5
	*A. baumannii*	03-149.01	5.9	7.0	7.6	7.9	8.1	8.5
	*A. baumannii*	AR #277	5.9	7.3	8.2	8.2	8.2	9.1
	*K. pneumoniae*	CRE-15	6.1	7.4	8.8	8.9	9.2	9.4
	*K. pneumoniae*	CRE-118	6.2	7.4	8.6	8.4	8.6	9.0
Controls for experiments that evaluate meropenem	*P. aeruginosa*	AR #250	6.2	7.0	8.1	8.7	8.9	9.5
	*A. baumannii*	N16870	5.4	6.6	7.7	8.4	8.4	8.6
	*K. pneumoniae*	CRE-15	5.9	6.7	8.4	8.5	8.6	8.6
	*K. pneumoniae*	CRE-118	6.0	7.7	8.6	8.8	8.9	9.2
Controls for experiments that evaluated cloxacillin	*S. aureus*	Clinical 2	5.9	7.1	8.5	8.7	8.9	9.2
Controls for experiments that evaluated levofloxacin	*S. aureus*	USA 300	6.1	7.1	8.2	8.7	8.8	9.1
